# A Classification Method of Point Clouds of Transmission Line Corridor Based on Improved Random Forest and Multi-Scale Features

**DOI:** 10.3390/s23031320

**Published:** 2023-01-24

**Authors:** Qingyun Tang, Letan Zhang, Guiwen Lan, Xiaoyong Shi, Xinghui Duanmu, Kan Chen

**Affiliations:** 1 College of Geomatics and Geoinformation, Guilin University of Technology, Guilin 541006, China; 2 Guangxi Key Laboratory of Spatial Information and Geomatics, Guilin University of Technology, Guilin 541006, China

**Keywords:** airborne laser scanning, improved Random Forest, multi-scale, feature selection, point cloud classification

## Abstract

Classification of airborne laser scanning (ALS) point clouds of power lines is of great importance to their reconstruction. However, it is still a difficult task to efficiently and accurately classify the ground, vegetation, power lines and power pylons from ALS point clouds. Therefore, in this paper, a method is proposed to improve the accuracy and efficiency of the classification of point clouds of transmission lines, which is based on improved Random Forest and multi-scale features. The point clouds are filtered by the optimized progressive TIN densification filtering algorithm, then the elevations of the filtered point cloud are normalized. The features of the point cloud at different scales are calculated according to the basic features of the point cloud and the characteristics of transmission lines. The Relief F and Sequential Backward Selection algorithm are used to select the best subset of features to estimate the parameters of the learning model, then an Improved Random Forest classification model is built to classify the point clouds. The proposed method is verified by using three different samples from the study area and the results show that, compared with the methods based on Support Vector Machines, AdaBoost or Random Forest, our method can reduce feature redundancy and has higher classification accuracy and efficiency.

## 1. Introduction

Like the arteries of the power grid, power lines are of great significance to economic development and the safety of people′s lives [1]. To ensure the safety and stability of power transmission, it is necessary to conduct regular inspections of the transmission corridor [2]. Due to the complex terrain of the transmission corridor, the traditional manual inspection method can no longer meet the needs of the modern smart grid. In recent years, the airborne LiDAR system has become an important tool for inspection of power lines, which can directly obtain aerial images of power lines and their ancillary equipment, as well as massive high-precision, high-density 3D point cloud data, providing a new means for power line inspection [3].

Presently, efforts in the inspection of transmission line corridors by using ALS point clouds include the four main following aspects: (1) point cloud classification of transmission lines [4,5]; (2) power line extraction [6,7,8,9]; (3) 3D reconstruction of power lines [10,11]; and (4) 3D reconstruction of power pylons [12,13]; whereas point cloud classification is the premise for other applications, such as 3D reconstruction of the transmission line corridors and the generation of digital surface models. Generally, point cloud classification includes four basic steps: data pre-processing, feature extraction, feature selection and classification. Many classification algorithms have been proposed. These algorithms can be divided into two classes according to the methods of feature extraction: (1) feature extraction by handcrafting [14,15]; (2) feature extraction by machine learning [16,17,18,19]. The methods of the first class establish a feature database by manually extracting feature parameters and the classification is conducted by matching the given features with the feature database. The classification results of these methods have a strong dependence on the selection and design method of artificial features. When building a feature database, a large number of models are required, resulting in a large labor workload and time cost for feature matching.

Point cloud classification methods based on machine learning mainly use Principal Component Analysis (PCA) feature description [20], point object-based feature representation [21], multi-dimensional feature histogram representation [22], waveform representation [23] and multi-scale feature representation [24,25] to describe and extract local and global features from point clouds. Classification is conducted with the feature parameters with strong descriptive capability by using machine learning classifiers such as Random Forest [4], JointBoost [5] and SVM [26].

Kim and Sohn [4] propose a point-based supervised classification method, which investigates a total of 21 features to illustrate the horizontal and vertical properties of power line objects, and a Random Forest model was trained with refined features to label raw laser point clouds. To further reduce misclassification, Guo et.al [5] proposed the use of spatial contextual information between objects in the transmission line corridor scenes for feature reduction. Feature reduction is conducted by using a Bayesian model of spatial correlation to optimize the decision structure of the JointBoost classifier. Wang Yanjun et.al [26] designed a framework for semi-automatic extraction of power lines with an SVM classifier. The candidate points of power lines are selected by a combination of the RANSAC algorithm and Hough transform [27] and 26 spatial structural features are selected to identify the point cloud of the transmission line corridor scene. Wang Pinghua et.al [28] proposed a method to extract power lines from the point cloud. The points for electric wires are roughly extracted according to the distribution characteristics of their elevations, the points for pylons are filtered with a Random Sample Consensus (RANSAC) algorithm [29] and the points for the insulators in the pylons are filtered with the statistical characteristics of their elevations. The resulting points are assigned to certain wires according to their elevations. However, with this method, when the points of the land surface are missing, the points for the power lines will be inclined to be incorrectly classified. 

In summary, feature extraction and selection is an important task for the above-mentioned classification methods based on machine learning. Currently, there are still some deficiencies when these methods are used to classify objects in complex scenes. The first is so-called feature redundancy when using multi-scale features. As the best neighborhood sizes for extracting different features are generally not the same, to enhance the adaptability of the feature parameters for various scenarios many features with weak correlation are also used, resulting in serious feature redundancy and low classification accuracy. The second is misclassification or omission of the adjacent objects at the boundary areas. The point clouds of certain objects are disordered in three-dimensional space, as the boundaries between adjacent objects are often not clear, therefore over-segmentation and under-segmentation often happen at the boundary areas, which results in misclassification or omission. Therefore, this paper proposes a point cloud classification method based on Random Forest, Relief F [30] and sequential backward selection [31] (REF-SBS).

(1) Ground points are separated by progressive triangulated irregular network (TIN) densification filtering. The neighborhood search is introduced to reduce the wrong classification of ground points. The point cloud elevation is normalized according to the optimized ground points to eliminate the negative impact of terrain relief.

(2) According to the ground features of the transmission corridor, the features of each point cloud are extracted with the neighborhood sizes of different scales to obtain the multi-scale feature set of the point cloud.

(3) Relief F and Sequential Backward Selection are used for feature evaluation and feature selection, respectively. The features with the best correlation are selected to train the model and realize the accurate classification of point clouds in transmission corridors.

The structure of this paper is as follows: Section 2.1 focuses on optimizing ground points by introducing a neighborhood search after filtering the point cloud and normalizing the point cloud data. Section 2.2 analyzes the point cloud features of the transmission corridor and explains the multi-scale feature set of the point cloud used in the experiment. In Section 2.3, the REF-SBS method is introduced into the random forest algorithm to select features and the selected features are used to train the model and classification. In Section 3, we provide an introduction to the data set used in our experiments. Section 4 presents the analysis and discussion of the experimental results. Finally, Section 5 concludes with a summary of our conclusions.

## 2. Method

**Figure 1:**
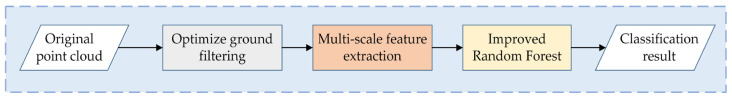
Overall workflow.

The proposed classification method mainly consists of three main steps, as shown in Figure 1: (1) Extracting ground points from the point cloud using the progressive TIN densification filtering and calculating normalized elevation of the point cloud with the ground points; (2) Construct the multi-scale geometric features of point clouds; (3) Classify the point cloud with the improved Random Forest algorithm. 

### 2.1. Ground Point Cloud Filter

A complex geographical environment and undulating terrain will cause huge differences in the elevations between feature points. When classifying the point clouds, it is easy to miss the ground points or misclassify the low feature points as the ground points [32]. This paper uses an optimized progressive TIN densification filtering to deal with the above problems. The basic steps of the method are shown as follows:

1. Remove noise points. Count the number of ALS points within the circular neighborhood of a certain point in 2D space; if the number of points is less than a set threshold, this point is considered a noise point and removed.
2. Select the lowest point within the divided point cloud grid as the initial ground point to construct the densified triangulated irregular network (TIN).
3. Optimize the filtered ground point cloud to increase the precision of classification, then the resulting points are normalized to be classified.

**Figure 2:**
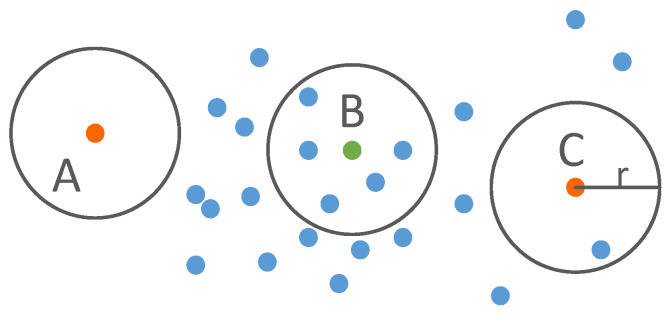
The principle of point cloud denoising.

Noise points are inevitably generated during the ALS scanning process. There are two types of typical noise points: (1) isolated points; (2) points below the average elevation of the land. When constructing a densified TIN, the existence of noise points will reduce the accuracy and reliability of the subsequent classification results, so it is necessary to remove noise points from the point cloud. Since there are often a very small number of points in the neighborhood of a noise point, it is reasonable to identify a noise point by comparing the number of points in its circular neighborhood with a pre-set threshold. As shown in Figure 2, Points A and C are identified as noise points, whereas Point B is not a noise point. 

**Figure 3:**
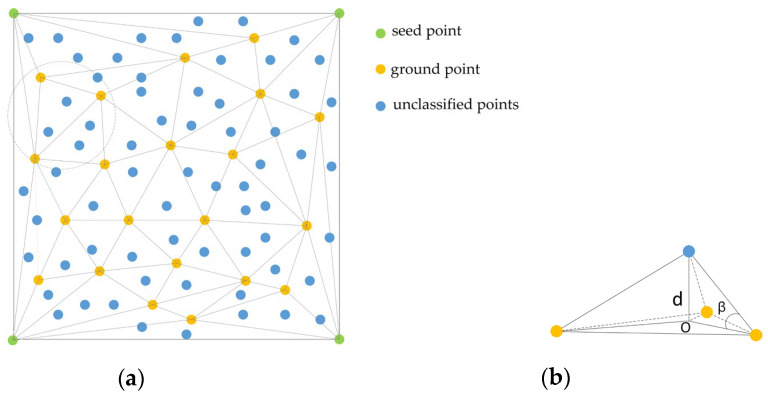
Determine whether an unclassified point is a ground point [32].

After denoising, the ground points are extracted by using progressive TIN densification filtering [32] and the basic idea is described as follows: (1) The point cloud is divided into a grid according to the maximum building size in the scene. (2) The lowest point in the grid is selected as the ground seed point, the other ground points are iteratively added by using the progressive TIN densification filtering and the iteration terminates when no other points are added to the TIN. The schematic diagram of the densified TIN is shown in Figure 3a. Figure 3b shows the densification process of a triangle of three ground points. In Figure 3b, the unclassified points are identified as ground points when iterative *d* and iterative *β* are less than the corresponding threshold. 

**Figure 4:**
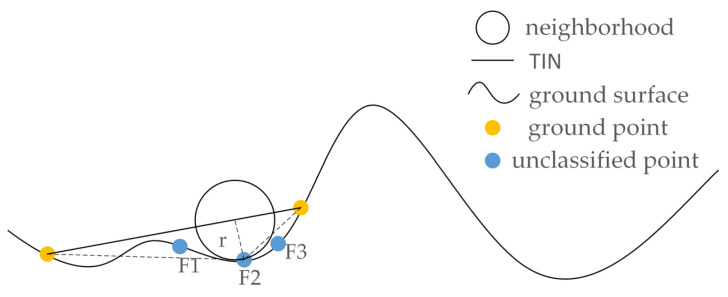
Determination of the misclassified ground points.

As some ground points may be lower than the plane of an identified ground triangle, the progressive TIN densification filtering may omit these ground points. In this paper, for a point in a triangle area, if its distance to the triangle is less than a pre-set threshold *r*, it is identified as a ground point, as shown in Figure 4. The extracted ground point clouds are used to generate a digital elevation model (DEM) by interpolation, then the other part of the point cloud (non-ground point) is normalized according to the DEM, to obtain the elevation features of the point clouds, which could help eliminate the influence of terrain undulation on the classification.

### 2.2. Multi-Scale Feature Extraction

The key to finding the most effective target classification method is to find the most effective features and develop the corresponding feature extraction methods [33]. ALS point clouds are commonly classified according to their basic features and geometric features. The basic features of point clouds include 3D coordinate information, intensity information, echo information and GPS time [34,35]. The echo information represents the penetration ability of the laser. The intensity information reflects to some extent the radiation force of the target [36]. The basic features are commonly unaffected by scale, rotation and illumination and are strongly robust [37]. The scale of geometric features will directly affect the classification accuracy of point clouds; therefore, for different scenes, it requires choosing the appropriate scale to extract features with high differentiation of categories in order to achieve accurate classification [38,39]. 

The geometric features of the point cloud can qualitatively represent the surface morphology of an object: a key element of the object structure, such as spatial distribution features, volume density, verticality, surface-related features, etc., can be obtained by statistical analysis and calculation of all points in the 3D neighborhood of a certain point [40,41]. 

The main geometric features include linearity ($L_{\lambda }$), planarity ($P_{\lambda }$), anisotropy ($A_{\lambda }$), spherical dispersion ($S_{\lambda }$) and the normal vector (N), volume density ($V_{ol}$), verticality ($V_{er}$), roughness ($R_{ou}$), and so on. For a point cloud in a certain neighborhood, its covariance matrix and eigenvalues can be calculated from the coordinates of the point cloud [41,42,43,44], then the geometric features are calculated with the covariance matrix and eigenvalues. Volume density ($V_{ol}$) represents the sparseness of points in the neighborhood of the point cloud. Verticality ($V_{er}$) represents the deviation of the local fit plane of the point cloud in the neighborhood from the horizontal plane. Roughness ($R_{ou}$) represents the standard deviation of the elevation of point clouds in the neighborhood. 

Owing to the uncertainty of the scene environment, multi-scale geometric features are usually used to classify objects from point clouds. To obtain the multi-scale geometric features, a list of spherical neighborhoods of different sizes centered on the target point are used to calculate the geometric features of the point cloud, respectively. For a spherical neighborhood, the geometric features are calculated by Equations (1) to (8).

(1) $$L_{\lambda }=(\lambda_{1}-\lambda_{2})/\lambda_{1},$$

(2) $$P_{\lambda }=(\lambda_{2}-\lambda_{3})/\lambda_{1},$$

(3) $$A_{\lambda }=(\lambda_{1}-\lambda_{3})/\lambda_{2},$$

(4) $$S_{\lambda }=\lambda_{3}/\lambda_{1},$$

(5) $$N=\lambda_{2}/(\lambda_{1}+\lambda_{2}+\lambda_{3}),$$

where $\lambda_{1}$, $\lambda_{2}$, $\lambda_{3}$ are eigenvalues, $L_{\lambda }$ is the linear feature, $P_{\lambda }$ is the planar feature,$A_{\lambda }$ is the anisotropy, $S_{\lambda }$ is spherical dispersion and N is the normal vector.

(6) $$V_{ol}=\frac{N_{R}}{V_{R}},$$

where $V_{ol}$ is volume density, $N_{R}$ is the number of point clouds in the neighborhood of the sphere with radius R and $V_{R}$ is the volume of the sphere with radius R.

(7) $$V_{er}=1-\left|Z\cdot N\right|,$$

where $V_{\mathrm{er}}$ is verticality, Z is the point cloud elevation and N is the normal vector.

The roughness of the target point cloud is calculated by fitting a plane to the point cloud through a least-squares adjustment. The standard deviation *σ* of the orthogonal residual distance *d* from all points to the fitted plane is chosen to numerically represent the roughness of the surface, as shown in Equation (8).

(8) $$R_{ou}=\sigma =\sqrt{\frac{1}{n}\sum_{1}^{n}{\left(d_{n}\right)}^{2}},$$

The scale of a geometric feature may decide its suitability in a certain classification operation, e.g., a feature of a small neighborhood can be suitable to recognize the boundary between different objects, while it is better to use a feature of a large neighborhood to recognize large objects. Therefore, it is of great importance to choose a suitable neighborhood for every geometric feature. In our study, for each geometric feature, 5 neighborhoods with different sizes, whose radii range from 1m to 8m, are used to calculate the feature values. The feature set containing 40 features is obtained. Then the best set of all features is input to the classifier for classification. As shown in Table 1, combined with the literature [5] and the characteristics of the transmission corridor, in our study, 8 geometric features are chosen to classify the point cloud into 5 categories.

### 2.3. The Improved Random Forest Algorithm Based on Relief F and SBS 

#### 2.3.1. The Related Algorithms 

Random Forest (RF) algorithm is an important ensemble learning method based on Bagging that can be applied to classification and regression problems [45]. The core idea of random forest is to combine multiple weak classifiers into a strong classifier with superior classification performance. The basic steps of point cloud classification using the RF algorithm are as follows: (1) Select training samples. (2) Build decision trees. (3) Generate the random forest model. (4) Classification.

Relief F (REF) is an algorithm for computing feature weights. Different weights are assigned to features according to the correlation between features. The features are ranked according to their weights. The features whose weight values are higher than the given weight threshold are selected as the feature subset. REF can handle incomplete and noisy data and address multi-class problems [30]. 

Sequential Backward Selection (SBS) is a method to automatically select a subset of features that are relevant to the problem. This method can improve the computational efficiency of the model and reduce the generalization error of the model by removing irrelevant feature parameters to eliminate noise [31]. The SBS algorithm can remove unimportant features from the input feature set and obtain the best feature subset through successive iterations.

#### 2.3.2. The Improved Random Forest Algorithm

**Figure 5:**
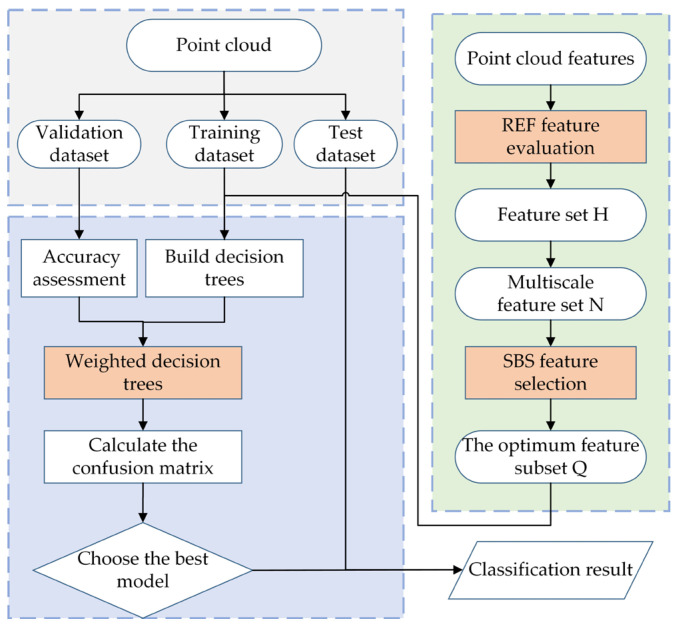
Improved Random Forest model.

Classical Random Forest is not effective in dealing with high-dimensional unbalanced data and the classification accuracy is relatively low. On the one hand, too high feature dimension will lead to serious redundancy and low computational performance of the algorithm. On the other hand, when processing unbalanced data, the predicted results tend to favor the majority vote, which affects the classification accuracy. To solve the above problems, a combination of REF and SBS is used in the proposed algorithm based on Random Forest. The improvements mainly include two aspects: (1) REF and SBS algorithms are used to optimize the selection of multi-scale neighborhood features. (2) By using weighted voting, the weight of the high-precision decision tree is modified to further improve the accuracy of point cloud classification, as shown in Figure 5.

The improved algorithm includes the following main steps:

(1) Evaluate the features of the point cloud with the Relief F algorithm. The feature weights of each sample in the training set are initially set to 0. For each feature, its weight is calculated with the method proposed in [30]. A sample *R* is randomly selected from the samples of the training set. Then, *i* samples with the same category as *R* are selected in the nearest neighbor domain of sample *R*. At the same time, *i* samples of different categories from *R* are selected in the nearest neighbor domain of sample *R*. The weight of the corresponding feature is calculated and finally the average value after *m* repetitions is used as the feature weight.

(2) Select the features from the multi-scale features with the SBS algorithm. Firstly, according to the ranking of the features, *h* features with strong correlation are selected to form the feature set H. The multi-scale feature set N is constructed by calculating the eigenvalues of each feature in different neighborhoods of the feature set H. Let *J* be the minimal standard measure function, which is used to represent the performance of classifiers. *J* is calculated before and after removing a feature, to determine which features to remove at each step. The features to remove at each stage are the ones that maximize the value of the function *J*, as shown in Equation (9).

(9) $$\bar{n}=\arg\max J(N_{k}-n),n\in N_{k}$$

where $\bar{n}$ is the feature to be deleted, $N_{k}$ is the initial feature set and *k* is the feature dimension. $N_{k-1}$ is the feature subset after removing features of Equation (10).

(10) $$N_{k-1}=N_{k}-\bar{n}$$

(3) Repeat step (2) to reduce the feature dimension until model performance loss is not acceptable, or when *k* reaches the preset number of features of the best feature subset Q.

(4) The feature subset Q is fed into the weighted Random Forest classifier for training. After random sampling, the random forest still has some out-of-bag (OOB) data. Therefore, the weight of the decision tree is evaluated based on its classification accuracy on the OOB data. The weighted voting principle is used to further improve the performance of the classifier. The weights are calculated as follows:

(11) $$W_{i}=\ln\frac{1+L_{i}}{1-L_{i}},\;i=1,2,\ldots ,n$$

where $W_{i}$ is the weight of the *i*th decision tree and $L_{i}$ is the correct classification rate of each decision tree by using OOB data.

(5) Classify the point clouds with the trained model. 

## 3. Datasets

**Figure 6:**
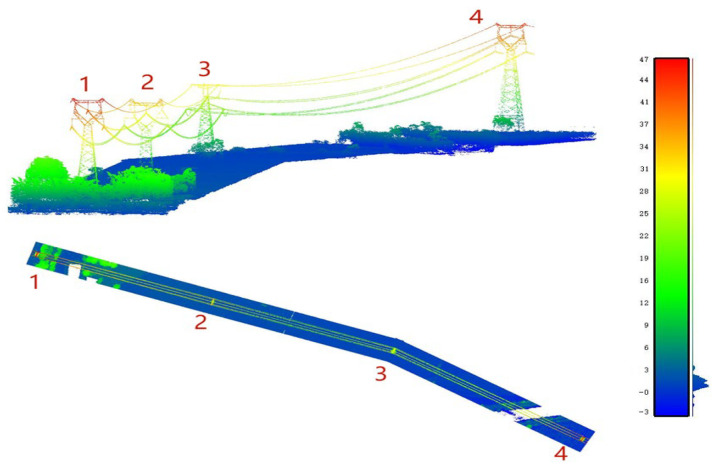
Power line point cloud.

The ALS data set used in our experiment was collected in Shaoguan, Guangdong, China. The format of point cloud data in the test area is LAS, including 3D coordinates of laser points, echo time, scanning angle and RGB information, as shown in Table 2. In addition to the ground points, the main ground objects in the experimental area include vegetation, power lines and power pylons. The original dataset is divided into three regions, 1–2, 2–3 and 2–4, and each region is labeled as A, B and C, respectively, as shown in Figure 6. Region A is used as the training set to train the model, while B and C are used as testing sets to test our proposed algorithm. Both the training and testing sets contain ground, vegetation, power lines, power pylons, etc. To analyze the correctness of the automatic classification of the proposed algorithm, we used CloudCompare to label the ground, vegetation, power lines and power towers. In addition, we manually classified the point clouds using the commercial software Terrasolid and used the classification results as the ground truth.

## 4. Results and Discussion

The main steps of the experiment include ground point cloud filtering, multi-scale feature extraction, feature selection and classification. With the Open CV image processing library and CloudCompare, the classification results of transmission line point clouds are visualized and analyzed. 

### 4.1. Results of Ground Point Cloud Filtering

**Figure 7:**
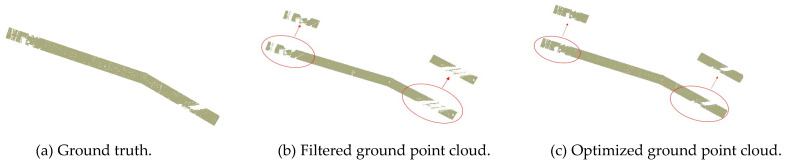
Extraction result of the ground point cloud.

**Figure 8:**
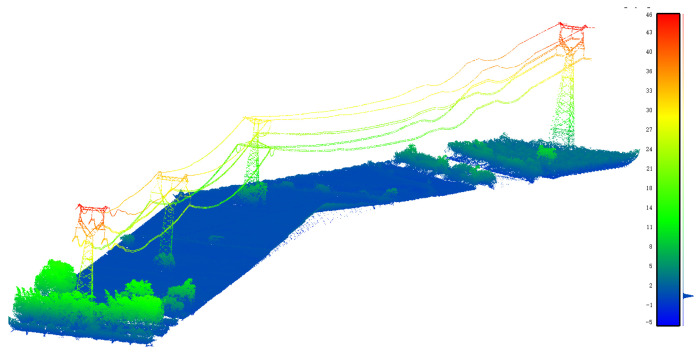
Normalized point cloud.

According to prior knowledge, we set the circular neighborhood radius to 5m and the quantity threshold to 2 when removing the noise points. This is a mountainous region, but the terrain is relatively flat and the main buildings in the region are power pylons. Therefore, when constructing the triangle network, we set the maximum slope to 88°, the iteration angle to 30° and the iteration distance to 0.8m. The ground point in this region is calculated by setting 0.05m as the vertical distance from the point to the TIN. It can be seen from Figure 7 that, compared with the ground points obtained after filtering, the optimized ground points are closer to the real ground truth, which further illustrates the necessity of optimizing the ground points. The transmission corridor point cloud is normalized according to the optimized ground points. The results are shown in Figure 8. 

### 4.2. Feature Extraction and Selection

**Figure 9:**
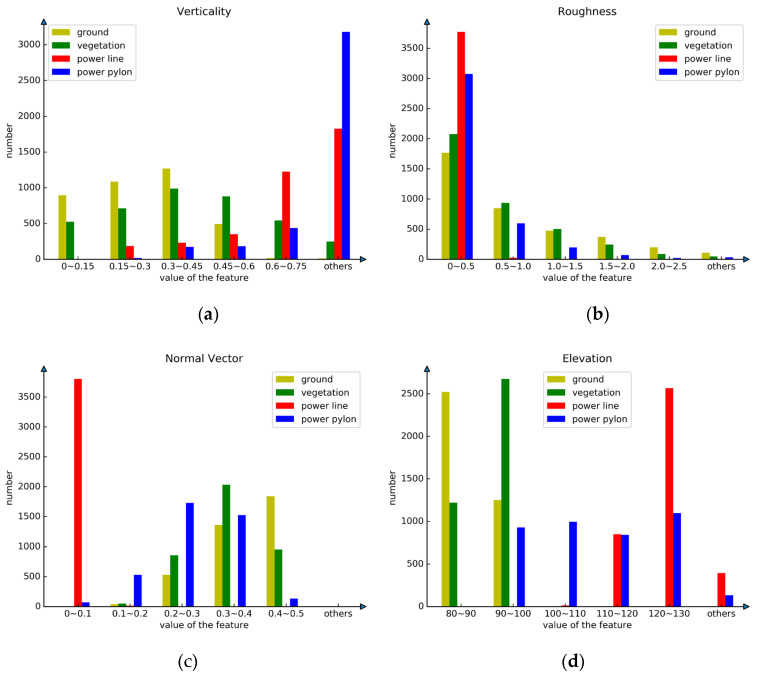

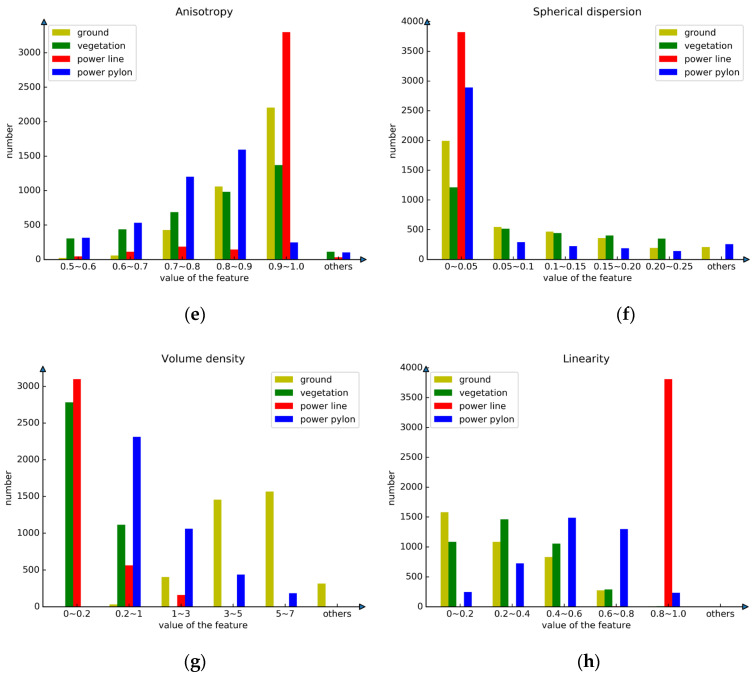
Comparison of point cloud features of four kinds of features.

The study area in this paper is a high-voltage transmission corridor located in a mountainous region. The category of ground objects in this region is relatively simple, but the distribution of ground objects varies greatly. The comparison of different ground features is shown in Figure 9, which shows that features such as verticality, normalized elevation, spherical dispersion, roughness, normal vector, anisotropy, linearity and volume density have different performances on different ground objects. The power lines in the transmission corridor have distinct linearity, elevation and normal vector. Verticality plays an important role in the classification of power pylons.

**Figure 10:**
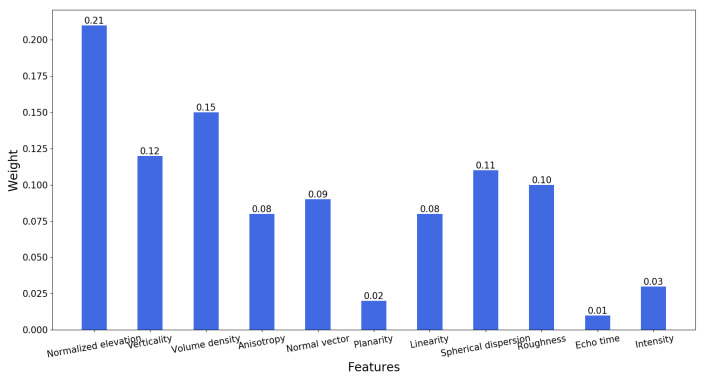
Feature weight evaluation.

In this paper, we use the REF algorithm to evaluate the feature weights for normalized elevation, verticality, volume density, anisotropy, normal vector, planarity, linearity, spherical dispersion, roughness, echo time and intensity, as shown in Figure 10. The weight of the normalized elevation is the largest, 0.21, and the weights of volume density, verticality, spherical dispersion, roughness, normal vector, anisotropy, linearity, intensity, planarity and echo time are 0.15, 0.12, 0.11, 0.10, 0.09, 0.08, 0.08, 0.03, 0.02, 0.01, respectively.

According to the ranking of feature weight evaluation, we select the top eight features by weight to calculate the multi-scale feature set. The top eight features are normalized elevation, verticality, spherical dispersion, roughness, normal vector, anisotropy, linearity and volume density. We use the selected neighborhood size in Section 2.2 to calculate point cloud features with neighborhood radii of 1 m, 2 m, 4 m, 6 m and 8 m, respectively, to obtain a multi-scale feature set containing 40 groups of features. 

**Figure 11:**
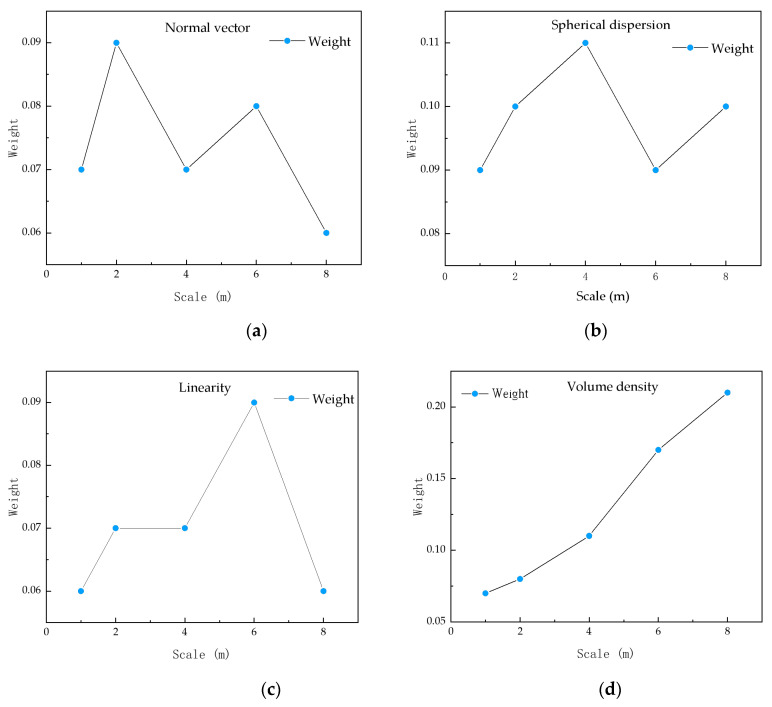

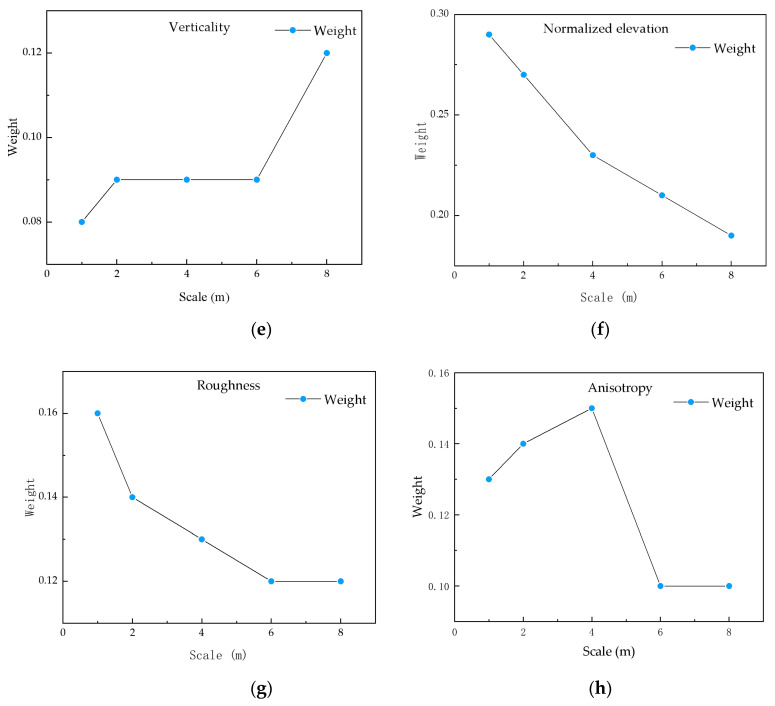
Multi-scale feature importance distribution.

The SBS algorithm is introduced to select the multi-scale feature set. According to the correlation between ground objects and features, we get the importance distribution of each feature at different scales. In Figure 11, the best neighborhood of eighty features, i.e., normalized elevation (*r* = 1 m), verticality (*r* = 8 m), roughness (*r* = 1 m), normal vector (*r* = 2 m), anisotropy (*r* = 4 m), linearity (*r* = 6 m) and volume density (*r* = 8 m), spherical dispersion (*r* = 4 m), is shown respectively. The eigenvalues at the best scale of each of the above features are used to obtain the best feature subset.

### 4.3. Classification Results of Transmission Line Point Clouds 

Random Forest is an ensemble learning algorithm that can be used to solve multi-class problems. The algorithm can effectively reduce the risk of over-fitting in the training. In this experiment, the dataset is divided into two parts: the training set and the testing set. The training part is used to build the model. Then, the model is evaluated through the testing set. All steps are implemented using the Python 3.7 programming language. The classification results are visualized using CloudCompare software.

In this paper, the classification results are evaluated using precision, recall and overall accuracy. The precision can be understood as the proportion of samples with correct predictions of those with positive predictions. The recall can be understood as the proportion of samples that are predicted correctly to those that are actually positive. Overall accuracy is the ratio of the number of correctly classified samples in a sample to the total number of samples. The defined Equations are shown in (12)–(14).

(12) $$\mathrm{Precision}=\frac{TP}{TP+FP},$$

(13) $$\mathrm{Recall}=\frac{TP}{TP+FN},$$

(14) $$\mathrm{Overall}\;\mathrm{Accuracy}=\frac{TP+TN}{TP+FN+FP+TN},$$

where *TP* (true positive), *TN* (true negative), *FP* (false positive) and *FN* (false negative), respectively, indicate the number of positive points that are correctly determined as positive, the number of negative points that are correctly determined as negative, the number of negative points that are incorrectly determined as positive and the number of positive points that are incorrectly classified as negative.

**Figure 12:**
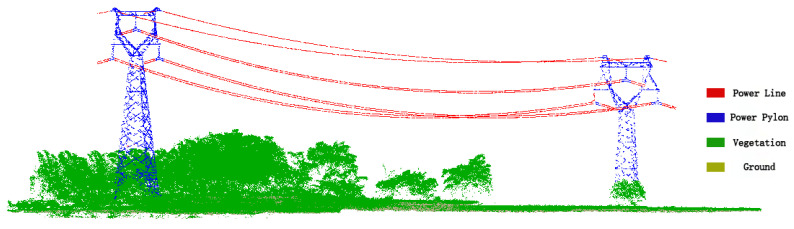
Point cloud in training region A.

**Figure 13:**
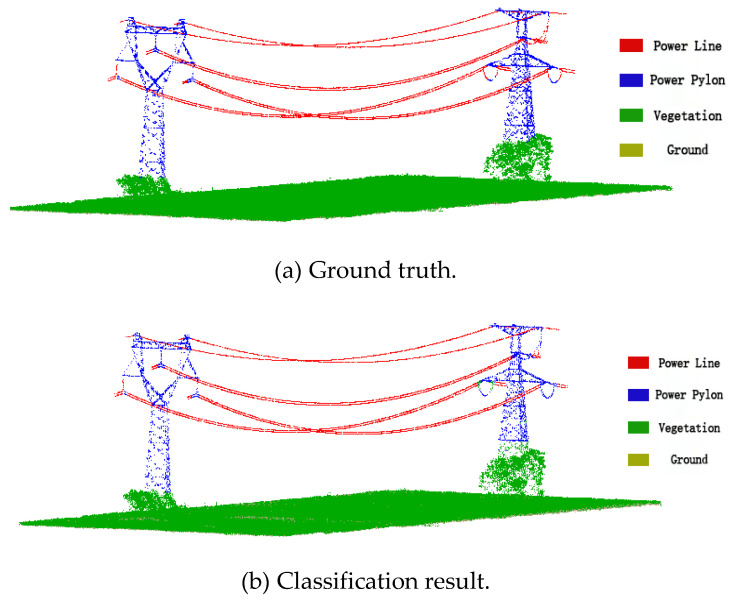
Classification result of region B point cloud.

**Figure 14:**
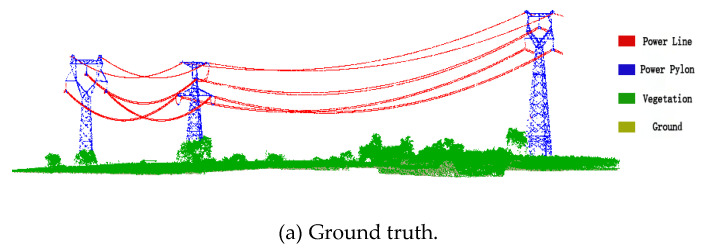

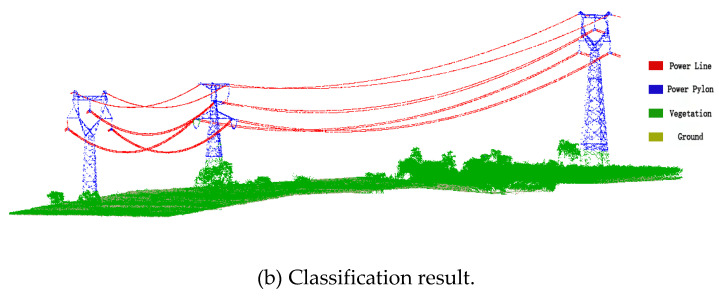
Classification result of region C point cloud.

The training set A is used to train the classification model, as shown in Figure 12. The verification set is divided from the training set A by the under-sampling method. The grid search method is used to adjust the model parameters and finally, parameter n_estimator of the model is 100, parameter max_depth of the model is 8. The trained classification model is then used to classify the testing sets B and C. The testing set has two regions: (1) region B with relatively flat terrain; (2) region C with undulating terrain. Figure 13 and Figure 14 show the classification results of regions B and C, respectively. 

The eight selected features are used for classification. Table 3 and Table 4 show the confusion matrices of regions B and C, respectively. The overall accuracy of regions B and C both reached 98%, indicating that the classification of the four categories of ground objects is basically correct. Misclassified points occur more frequently in the ground class and they are misclassified as vegetation. The reason is that the height of some vegetation is too low to accurately distinguish the ground from the vegetation. In the power pylon category, some points at the bottom of the power pylon are misclassified as vegetation. Because the vegetation distribution is relatively dispersed, the volume density of a small part of the vegetation is sparse, which is similar to the volume density and roughness of the bottom of the power pylon. In the power line category, some power line points are identified as power pylon points, because the power pylon is a trapezoidal or triangular steel frame structure. From the side view of the power pylon, the power pylon has similar linear features as the power line.

To verify the effectiveness of the method in this paper, RF [4], AdaBoost [16] and SVM [26] algorithms are used to classify the point cloud of the transmission corridor. The comparison table of classification results is shown in Table 5. In terms of overall accuracy, that of the improved random forest algorithm is significantly higher than that of the other three algorithms. From the classification precision of each category, the improved random forest has obvious advantages in the classification of ground points. Compared with the other three classification algorithms, the improved random forest improves the precision of ground points by more than 10%. According to the classification results of power pylons, the precision of the proposed algorithm is 94.47%, which is also significantly higher than that of SVM, AdaBoost, and RF. In terms of the classification efficiency of the model, due to a large amount of point cloud data, SVM needs to perform matrix calculation during classification, which consumes more time. However, the improved Random Forest greatly improves the classification efficiency and can further handle large-scale point clouds.

### 4.4. Discussion 

Since the point cloud is continuously distributed, the neighborhood of different sizes directly affects the classification results. Therefore, it is necessary to consider the coordination of point cloud features at different scales. To explore the classification of point clouds in transmission corridors, we construct a multi-scale feature set containing 40 features according to the spatial location of point clouds in different neighborhood sizes. The feature set includes eight main kinds of point cloud geometric features in five different neighborhood sizes. When performing a classification task, the higher the feature dimension, the more redundant information and the greater the optimization for dimension reduction [46]. To ensure the best performance of the classifier, it is necessary to select the features. Therefore, a REF-SBS feature selection algorithm is used to select multi-scale features of point clouds. According to the result of feature selection, this method can effectively select the features with obvious differences among various features in the transmission corridor. The classification accuracy and efficiency are improved to a certain extent. We take the result of feature selection as the best feature subset, use the best feature subset for training and use the trained model to classify the test set. The classification results of the test set show the effectiveness of the proposed method in point cloud classification of transmission corridors.

## 5. Conclusions

In this paper, we propose an improved random forest classification algorithm for point cloud classification of transmission corridors. The experimental results show that this method can reduce the feature dimension in point cloud classification, choose the features with a strong correlation with the category and improve the accuracy and efficiency of point cloud classification. The advantages of the proposed method can be summarized as follows:

(1) The neighborhood search method is introduced to optimize the filtered ground point cloud, which can improve the classification accuracy of ground points. Combined with the optimized ground point cloud, the transmission line point cloud is normalized to eliminate the influence of terrain on the point cloud classification results.

(2) Multi-scale features can reflect the differences of ground objects in different neighborhood sizes. By analyzing the characteristics of transmission corridors, a multi-scale feature set is constructed to ensure classification accuracy.

(3) The REF-SBS algorithm is used to weight the features with a strong correlation to the category. Features that are weakly correlated with the category are removed. This process can reduce feature redundancy and improve the classification accuracy of point clouds.

Point cloud classification is a complex and challenging task. At present, the inspection of transmission corridors based on UAV photogrammetry system is widely used. In future work, we will fuse the ALS point cloud data with the photogrammetric image data to further improve the applicability of our proposed method.

## Figures and Tables

**Table 1:** The geometric features used in classification of point clouds for transmission line corridor.

Category	Point Cloud Features	Scales
ground	Linearity ($L_{\lambda }$) Planarity ($P_{\lambda }$) Anisotropy ($A_{\lambda }$) Spherical dispersion ($S_{\lambda }$) Normal vector (N) Volume density ($V_{ol}$) Verticality ($V_{er}$) Roughness ($R_{ou}$)	1 m 2 m 4 m 6 m 8 m
building		
vegetation		
power line		
power pylon		

**Table 2:** Information for the experimental dataset.

Dataset	Area (m^2^)	Density (pt/m^2^)	Number of Points
Training set A	331 × 52	79	1,362,684
Testing set B	342 × 52	65	1,158,634
Testing set C	721 × 52	64	2,411,158

**Table 3:** Confusion matrix for classification of region B.

Overall Accuracy: 98.73%					
Category	Ground	Vegetation	Power Line	Power Pylon	Recall/%
**ground**	30,703	8086	0	0	80.18
**vegetation**	6015	1,101,805	0	282	99.43
**power line**	0	0	7220	279	96.27
**power pylon**	0	228	265	7851	94.09
**Precision/%**	83.61	99.25	96.45	93.33	

**Table 4:** Confusion matrix for classification of region C.

Overall accuracy: 99.1%					
Category	Ground	Vegetation	Power Line	Power Pylon	Recall/%
**ground**	57,914	10,304	0	0	84.89
**vegetation**	9811	2,302,050	50	40	99.57
**power line**	0	15	15,950	754	95.4
**power pylon**	0	328	619	13,323	93.36
**Precision/%**	85.51	99.53	95.97	94.38	

**Table 5:** Comparative analysis of different classification methods.

Classifier	Precision/%				Overall Accuracy/%	Time/s
	Ground	Vegetation	Power Line	Pylon		
RF [4]	77.26	97.14	96.38	89.41	96.14	192
AdaBoost [16]	72.41	95.98	96.16	87.85	93.91	920
SVM [26]	73.21	97.32	82.15	88.39	95.93	2290
Improved RF	88.39	99.10	97.25	94.47	98.20	96

## Data Availability

The data used to support the findings of this study are available from the corresponding author upon request.
